# The management of congenital adrenal hyperplasia during preconception, pregnancy, and postpartum

**DOI:** 10.1007/s11154-022-09770-5

**Published:** 2022-11-18

**Authors:** Jacqueline Yano Maher, Veronica Gomez-Lobo, Deborah P. Merke

**Affiliations:** 1 Eunice Kennedy Shriver National Institute of Child Health and Human Development, Staff Clinician, 10 Central Drive, Room 8N248, Bethesda, MD 20892 USA; 2 Director of Pediatric and Adolescent Gynecology, Eunice Kennedy Shriver National Institute of Child Health and Human Development, 10 Central Drive, Room 8N248, Bethesda, MD 20892 USA; 3 National Institutes of Health Clinical Center and Eunice Kennedy Shriver National Institute of Child Health and Human Development, 10 Central Drive, Room 1-2740, Bethesda, MD 20892 USA

**Keywords:** Congenital adrenal hyperplasia, 21-hydroxylase deficiency, Contraception, Fertility, Pregnancy

## Abstract

Congenital adrenal hyperplasia (CAH) is a group of autosomal recessive disorders of steroidogenesis of the adrenal cortex, most commonly due to 21-hydroxylase deficiency caused by mutations in the *CYP21A2* gene. Although women with CAH have decreased fecundity, they are able to conceive; thus, if pregnancy is not desired, contraception options should be offered. If fertility is desired, women with classic CAH should first optimize glucocorticoid treatment, followed by ovulation induction medications and gonadotropins if needed. Due to the possible pregnancy complications and implications on the offspring, preconception genetic testing and counseling with a high-risk obstetrics specialist is recommended. For couples trying to avoid having a child with CAH, care with a reproductive endocrinology and infertility specialist to utilize *in vitro* fertilization can be offered, with or without preimplantation genetic testing for monogenic disorders. Prenatal screening and diagnosis options during pregnancy include maternal serum cell free-DNA for sex of the baby, and chorionic villus sampling and amniocentesis for diagnosis of CAH. Pregnant women with classic CAH need glucocorticoids to be adjusted during the pregnancy, at the time of delivery, and postpartum, and should be monitored for adrenal crisis. Maternal and fetal risks may include chorioamnionitis, maternal hypertension, gestational diabetes, cesarean section, and small for gestational age infants. This review on CAH due to 21-hydroxylase deficiency highlights reproductive health including genetic transmission, contraception options, glucocorticoid management, fertility treatments, as well as testing, antenatal monitoring, and management during pregnancy, delivery, and postpartum.

## Introduction

Congenital adrenal hyperplasia (CAH) is a group of autosomal recessive disorders of steroidogenesis of the adrenal cortex. It is most commonly due to 21-hydroxylase deficiency caused by mutations in the *CYP21A2* gene [1]. Other causes of CAH include deficiencies of: 11β-hydroxylase, 3β-hydroxysteroid dehydrogenase, 17α-hydroxylase/17–20 lyase, steroidogenic acute regulatory protein (StAR), cholesterol side chain cleavage enzyme, and cytochrome P450 oxidoreductase [2]. This review will focus on CAH due to 21-hydroxylase deficiency.

**Fig. 1:**
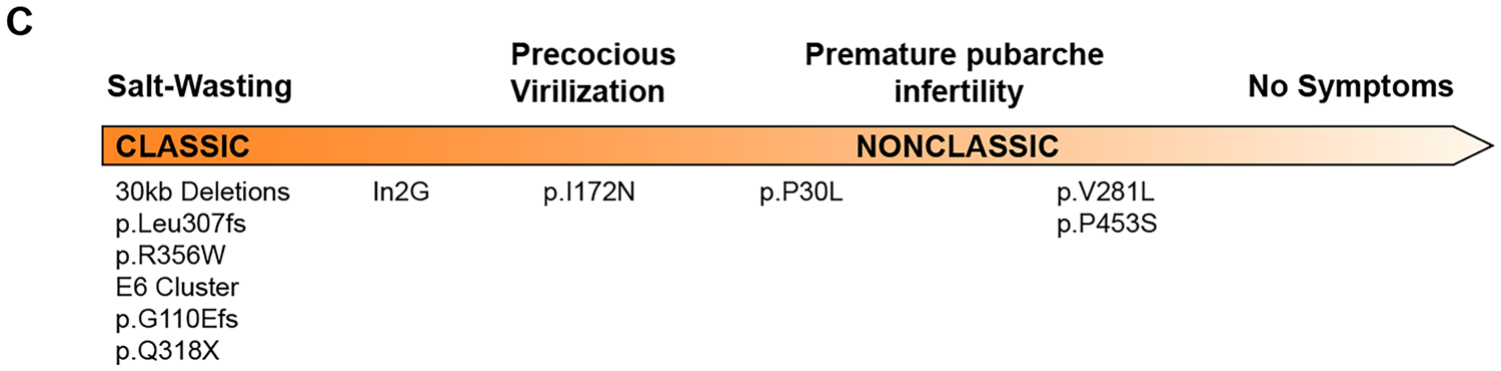
The genetics of congenital adrenal hyperplasia due to 21-hydroxylase deficiency. The most commonly found mutations are shown in relation to the phenotypic spectrum. Mutations range from complete loss of function of the 21-hydroxylase enzyme associated with the classic salt-wasting type to mild impairment with 30 to 50 percent of enzyme activity associated with the nonclassic type. Overall, there is a continuum of disease severity with clinical overlap of these categories, good correlation between genotype and phenotype for severe mutations (residual 21-hydroxylase function 0–1%) and greater phenotype variability amongst intermediate and less severe genotypes. *Adapted from* Merke et al. *N Engl J Med* 2020

Deficiency of 21-hydroxylase results in multiple hormonal imbalances including cortisol and aldosterone deficiencies and androgen excess. CAH in its classic (severe) form is part of neonatal screening programs in the US and over 50 other countries [3]. Based on neonatal screening, the classic form occurs in 1:14,000 to 1: 18,000 worldwide [3]. Approximately 75 percent of patients with the classic form have severely reduced or absent 21-hydroxylase enzyme activity and clinically present with a potentially fatal salt-wasting adrenal crisis in the neonatal period if treatment is not promptly initiated. These patients make up the classic salt-wasting (SW) subtype. The remaining 25 percent of classic patients have *CYP21A2* mutations associated with residual 21-hydroxylase activity of 1 to 5%. These patients produce small amounts of aldosterone, are less likely to suffer an acute salt-wasting crisis in the newborn period, and have been termed the classic simple virilizing (SV) subtype. The nonclassic (mild) form may be asymptomatic and is quite common with an estimated prevalence of 1 in 200 in Caucasians in the US [4]. Although historically classic and nonclassic genotypes and phenotypes have been described, there is a continuum of disease severity and phenotypic variations occur (Fig. 1). In general, there is good genotype–phenotype correlation for mutations with little residual 21-hydroxylase activity (< 1%), and greater phenotype variability with intermediate and less affected genotypes.

In the adrenal cortex, impairment of the 21-hydroxylase enzyme results in decreased cortisol and aldosterone production. Reduced cortisol results in lack of negative feedback on the hypothalamic–pituitary–adrenal axis resulting in increased pituitary corticotropin production. The combination of excess corticotropin and accumulation of precursor steroids prior to the 21-hydroxylase enzymatic block results in increased adrenal androgen production. In the classic type, markedly increased prenatal adrenal androgen virilizes the 46,XX external genitalia, and classic CAH due to 21-hydroxylase deficiency is the most common cause of atypical genitalia in the newborn [2]. First trimester exposure to excess androgen results in formation of a urogenital sinus with a conjoined urethra and vagina, and further exposure to androgens throughout pregnancy results in variable degrees of labial fusion and clitoral enlargement. The uterus, ovaries and fallopian tubes are normal. The routine practice of genital surgery in infancy has been questioned, and shared decision making among parents, patients, surgeons, endocrinologists, and mental health providers is currently promoted [5, 6]. Standard treatment of classic CAH involves lifetime glucocorticoid and often mineralocorticoid replacement, and adequate control of adrenal androgen production usually requires supraphysiologic glucocorticoid therapy. Specialists caring for patients with CAH should consider general psychosocial issues (i.e. mental health challenges, coping with a chronic illness) and CAH-specific sequelae (i.e. stigma associated with the classic form and fertility concerns) [3, 7, 8].

Although patients with classic CAH survive the disease as a result of neonatal screening and early treatment, existing therapies have failed to prevent multiple comorbidities due to hyperandrogenism, iatrogenic glucocorticoid excess, or a combination of these two undesirable states. Prolonged periods of excess adrenal androgens places children at risk of central precocious puberty and halted breast development, and women may experience acne, hirsutism, irregular menses, and impaired fertility [9–11]. Adrenal-derived progesterone also accumulates and impedes conception. Compared to unaffected women, women with classic CAH have fewer pregnancies and fewer children [12] and are less likely to seek motherhood [13].

In general, there is an association between the severity of the CAH genotype and the degree of ovarian dysfunction and infertility [3, 14]. Females with nonclassic CAH (NCCAH) are born with typical female genitalia, sometimes present during childhood with premature pubarche, may not be diagnosed until they are adults during a work-up for infertility, or may be asymptomatic [15]. Women with NCAH have excess adrenal androgens and progesterone, similar to women with the classic form, but to a lesser extent.

When patients with classic or NCCAH do attempt pregnancy, the success rate has increased in the last two decades due to improvements in our understanding of the effect of androgen and progesterone levels on female infertility and the importance of optimal glucocorticoid and sometimes mineralocorticoid replacement. This review summarizes the management of CAH during preconception, pregnancy, and postpartum.

## Contraception

Although women with CAH have decreased fecundity, they are able to conceive. Thus, it is important to assess their contraceptive needs and desires. There is a wide number of options for contraception to date with some having specific implications for women with CAH. For those with comorbidities, a useful resource for clinicians is the U.S. Medical Eligibility Criteria for Contraceptive Use [16].

Long-acting reversible contraception including intrauterine devices (copper or hormonal) and contraceptive implants are associated with very low pregnancy rates as they do not depend on regular user compliance [16]. However, women with CAH may encounter difficulties with insertion of the intrauterine device (IUD) due to vaginal stenosis. Progesterone only methods include progesterone pills, injections (Depo-Provera), levonorgestrel implant (Nexplanon) and IUD. Women with CAH may experience menstrual irregularity and thinning of the endometrium due to elevated progesterone which may be associated with infertility [17]. Adding a progesterone contraceptive method when a patient wants to assure pregnancy prevention, however, should be safe and effective. Of note, the Depo-Provera injection is associated with decrease in estrogen and reversible decreases in bone mineral density an important implication in women with CAH given their treatment with corticosteroids [16]. In addition, in some populations Depo-Provera is associated with significant weight gain, another issue that may impact women with CAH differently than the general population. Combined hormonal contraception (birth control pills, contraceptive ring and patches) contain estrogen which is associated with higher risk of complications including venous thromboembolism and hypertension [16]. These, however, can assist with control of menses as well as decrease the androgenic symptoms of CAH. Benefits for the treatment of acne and hirsutism are due to increases in sex hormone binding globulin due to estrogen effects on the liver. Drosperinone, a progesterone derived from spironolactone, is available both in combined as well as progesterone only pills and may further improve symptoms of acne and hirsutism. However, drosperinone may also have some mineralocorticoid antagonistic properties thus possibly affecting the dose of fludrocortisone required, but this has not been studied.

Barrier methods such as female condom, sponge, diaphragm, and cervical cap may be difficult for a woman with classic CAH to insert, while male condoms do not present these limitations and also provide protection from sexually transmitted infections. Finally, hormonal post-coital contraception pills have few side effects, and some can be accessed over the counter. Ultimately, it is important that the clinician discuss each patient’s priorities when counseling about contraception and allow for shared decision making [18] (Table 1).

**Table 1:** Contraceptive Methods and Considerations in Women with Congenital Adrenal Hyperplasia (CAH)

**Method Type**	**Risks/Side Effects**	**Considerations for CAH**
Intrauterine device (Copper)*	Heavy menses	Intrauterine device may be difficult to insert in classic CAH
Intrauterine device (Levonorgestrel)*	Irregular bleeding	Intrauterine device may be difficult to insert in classic CAH
Contraceptive implant – arm (Nexplanon)*	Irregular Bleeding	
Depo-Provera (DMPA) – Intramuscular injection	Irregular bleeding Reversible decrease in bone mineral density Possible weight gain	Amplified risk of adverse effects on bone mineral density and weight gain
Progestin-only pills	Breakthrough bleeding with Norethindrone pill	Drosperinone pill may improve acne and hirsutism
Combined hormonal contraception (pill, patch, ring)	Increased risk of venous thromboembolism and hypertension	Can regulate menses Can improve acne and hirsutism

*Long-acting reversible contraception (LARC)

## Fertility and reproduction

### Preconception counseling

**Fig. 2:**
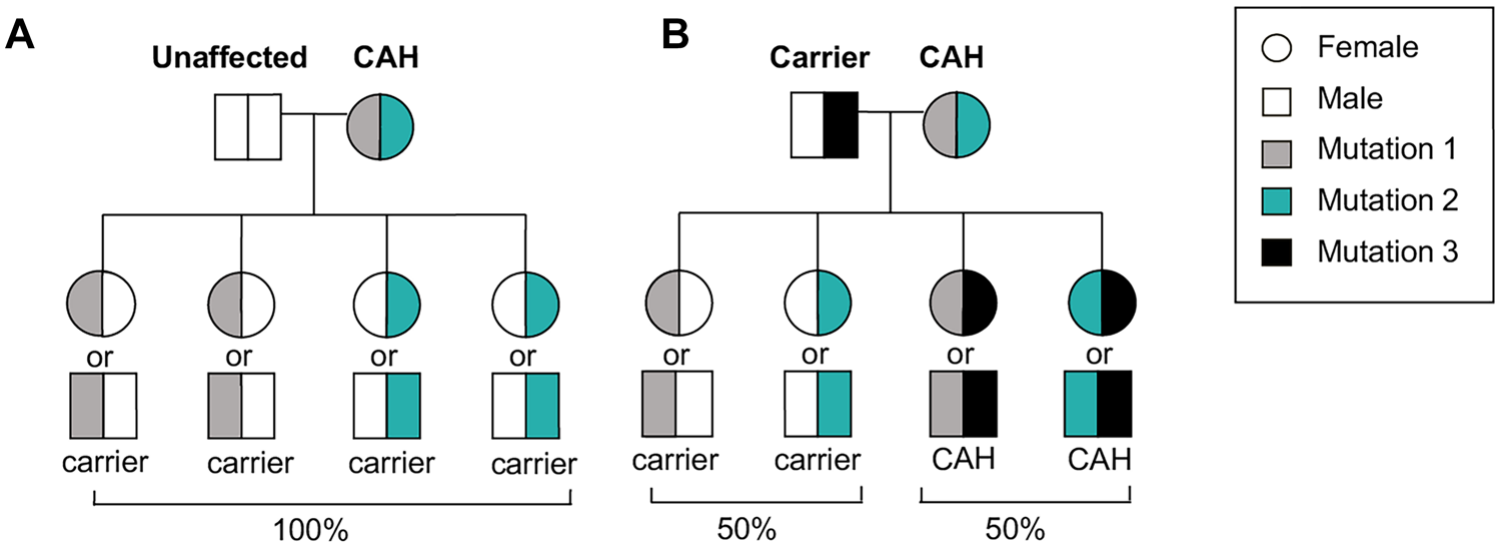
Genetic risk of a patient with CAH having a child with CAH. The majority of patients with CAH are compound heterozygotes with different mutations on each allele and a phenotype corresponding to the milder gene defect. (**A**) If the partner of a patient with CAH is not a carrier, then all offspring will be carriers. (**B**) If the partner of a patient with CAH is a carrier of CAH, then there is a 50 percent risk of CAH in the offspring. Based on the incidence of classic CAH, approximately 1 in 60 individuals carry an allele that causes classic CAH. Therefore, the probability that a patient with classic CAH will have a child with classic CAH is 1 in 120 (1/60 x ½) if the genotype of the partner is unknown. Genetic counseling and genotyping are recommended prior to conception

**Fig. 3:**
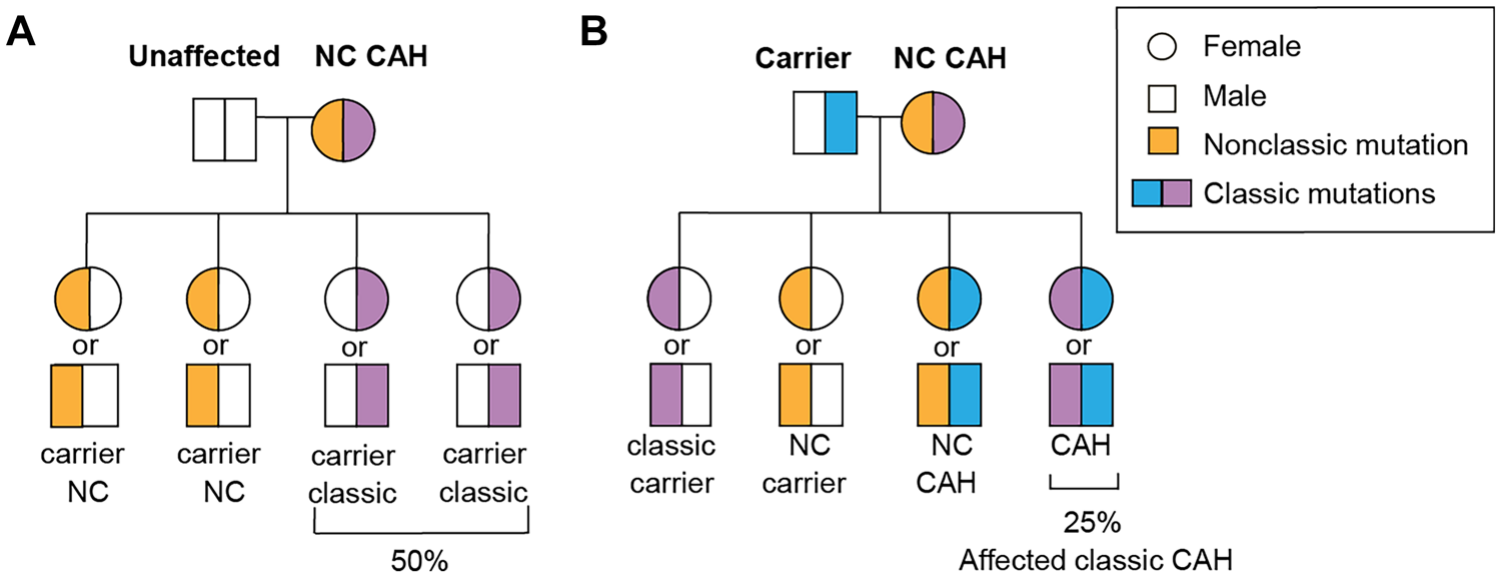
Genetic risk of a patient with nonclassic CAH (NCCAH) having a child with classic CAH. (**A**) Approximately two-thirds of patients with NCCAH carry an allele that causes classic CAH. Therefore, half of all children might be carriers of a classic gene. (**B**) If the partner of a patient with NCCAH is a carrier of classic CAH and the patient with NCCAH carries an allele that causes classic CAH, then there is a 25 percent risk of classic CAH and 25 percent risk of NC CAH in offspring. If the genotypes are unknown, the risk of a patient with NCCAH having a child with classic CAH is approximately 1 in 350 (2/3 × 1/2 × 1/60 × 1/2), but a higher prevalence up to 2.3% has been shown in retrospective cohort studies [21, 22]

Due to the possible pregnancy complications and implications on the offspring, preconception genetic testing and counseling with a high risk obstetrics specialist is recommended in women with a known diagnosis of classic CAH who are family planning [19, 20]. The carriership in the general population for classic CAH is approximately 1 in 60 (between 1 and 2 percent) [3]. Therefore, if the carriership of the partner is unknown, the risk of having a child with classic CAH is approximately 1 in 120 (Fig. 2). However, if the partner is a known carrier of classic CAH, then the risk of having an affected child is 50 percent. The majority of patients with NCCAH are compound heterozygotes with different mutations on each allele, and approximately two-thirds carry one allele that causes classic CAH (Fig. 3). [21] Theoretically and without genotyping, a patient with NCCAH has a 1 in 350 risk of having a child with classic CAH. An international multicenter study of 203 pregnancies in 101 women with NCCAH reported a much higher risk of 2.5% of conceiving a child with classic CAH and 14.8% with NCCAH [22]. Similarly, a retrospective study of children born to 190 women with NCCAH in France estimated the risk of having a child with classic CAH to be 1.5% [21]. The higher than expected rate of classic CAH in these studies may be due to the high frequency of marriages between people of similar ethnicities resulting in a higher likelihood of those that are carriers intermarrying. The estimated carrier frequency of NCCAH varies from 1:7 to 1:16, and the epidemiology of NCCAH is less well established than classic CAH because it is not detected by neonatal screening programs [4]. Therefore, *CYP21A2* genotyping is recommended prior to pregnancy to better define individual risk. Routine genetic carrier screening, which includes CAH due to 21-hydroxylase deficiency, is becoming more affordable and is recommended by the American College of Obstetrics and Gynecology to be offered to all pregnant patients and those desiring pregnancy during preconception visits [23], especially if undergoing an infertility work up and *in vitro* fertilization (IVF).

### Fertility and classic CAH

Compared to the general population, women with classic CAH are less likely to seek motherhood and have fewer pregnancies and children [13]. In a population-based study using the national Swedish registry, only 25.4% of women with CAH had given birth compared with 45.8% of controls [24]. Interestingly, the reduction in motherhood in CAH was only observed in women with SW CAH, while those with SV and NCCAH were similar to matched controls. In the UK, the pregnancy rate of women with CAH was similar to the general UK population, however women with SW CAH were less likely to seek motherhood [12]. This is consistent with other studies that report fewer pregnancies and less desire for fertility in women with the SW phenotype [14]. There are a multitude of potential causes including possible prenatal androgen effects on the brain and behavior [25], an increased prevalence of gender identity shifts in sexual orientation and reduced likelihood to be exclusively heterosexual compared to unaffected women [26–28], hormonal imbalances leading to anovulation, and decreased sexual satisfaction due to urogenital dysfunction [28, 29].

Women with CAH have multiple hormonal imbalances contributing to subfertility. Androgen excess suppresses late stage folliculogenesis and ovulation. Preferential secretion of LH further increases androgen levels and elevated androgens are aromatized to estrogens and both suppress gonadotropin secretion. High progesterone levels impact fertility on many levels; it disrupts GnRH pulsatility and ovulation, thickens cervical mucus and interferes with sperm motility, and impacts endometrial lining growth which impairs embryo implantation [9]. However, with glucocorticoid and mineralocorticoid treatment, menstrual cycles can become ovulatory about 40% of the time [30]. Fertility can be restored in the majority of women, with natural conception reported as high as 76% [12]. However, in a European (6 countries: Germany, France, the Netherlands, Poland, Sweden and United Kingdom) multicenter cross-sectional study of disorders of sex development, only 14.7% of 221 women with CAH had one or more children without assisted reproduction techniques [31]. Numbers of oocytes appear to not be affected in patients with CAH, as indicated by a study evaluating anti-mullerian hormone (AMH, a marker of ovarian reserve) that found no significant difference between CAH patients and controls and comparable levels between classic and NCCAH patients [32].

CAH has a spectrum of anatomic presentations due to the varied virilizing effects of androgen exposure to the external genitalia. Furthermore, surgical management may further impact anatomy and function. Anatomic factors that may interfere with fertility include a smaller vaginal introitus, decreased vaginal lubrication, dyspareunia, decreased clitoral sensitivity secondary to surgery, and decreased sexual satisfaction [33–35]. In a meta-analysis of 29 studies, most patients were sexually active, but only 48% reported comfortable sexual intercourse [36]. These negative experiences may make intercourse less desirable and therefore decrease the chances of conception based on frequency and timing.

### Fertility and nonclassic CAH

Women with NCCAH are at risk for spontaneous miscarriage, which has been reported in up to 25% of pregnancies and shown to be reduced with glucocorticoid treatment. [21, 22, 37] If impaired fertility occurs, prednisone or hydrocortisone can be used, and dexamethasone should be avoided since it can cross the fetoplacental barrier and may impact cognitive development [38, 39]. However, there are no clear recommendations regarding the treatment of NCCAH during pregnancy [40]. Similar to polycystic ovary syndrome, which NCCAH is sometimes misdiagnosed as, the main cause of infertility is anovulation in 30–50% of patients [22, 41]. This results in complaints of subfertility in 10–30% of women with NCCAH seeking care [21, 22]. Glucocorticoid therapy is recommended for patients with NCCAH and infertility (no conception after 12 months) or history of miscarriage. Though it is reasonable to refer to a reproductive endocrinology and infertility (REI) specialist if no conception after 6 months.

### Treatment of infertility in classic and NCCAH

**Fig. 4:**
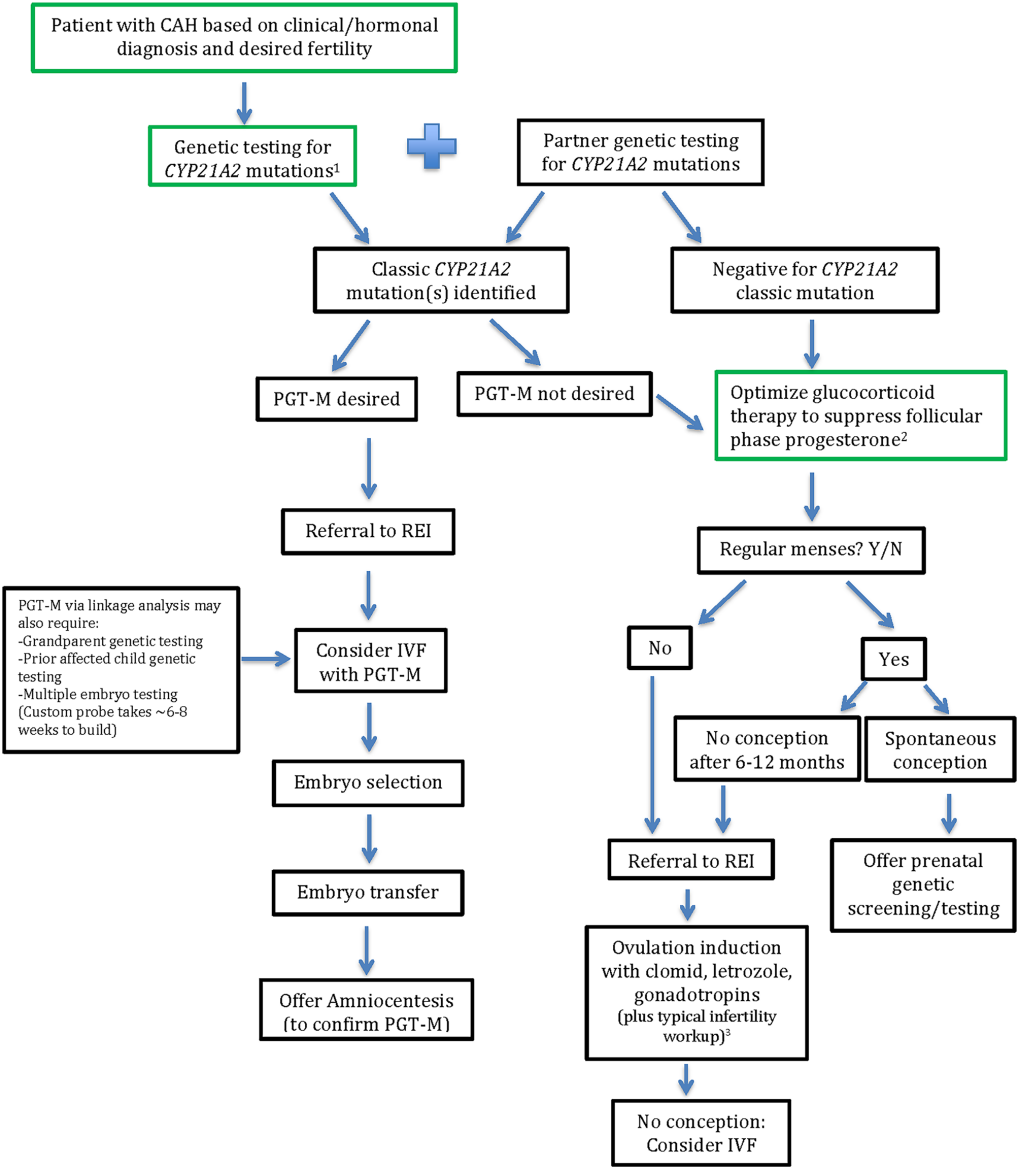
Preconception and infertility management for couples who desire fertility. Many patients with CAH may only have a clinical diagnosis. In order to identify and minimize the risk of having a child affected with CAH, partner genetic testing is recommended, especially if the partner is from the same family or ethnicity. Green boxes show treatment for the female patient with CAH. ^1^Approximately two-thirds of patients with NCCAH carry an allele that causes classic CAH (see Fig. 3). Both alleles of patients with classic CAH carry mutations that cause classic CAH. ^2^Glucocorticoid therapy is recommended for patients with NC CAH and infertility (no conception after 12 months) or history of miscarriage. ^3^Ovarian reserve testing, semen analysis, hysterosalpingogram/sonohystogram

The first goal in addressing infertility in women with classic CAH is to optimize glucocorticoid treatment in an attempt to achieve regular ovulation and menstruation [5] (Fig. 4). Hydrocortisone, prednisone, or prednisolone can be given, which is preferred over dexamethasone which crosses the placenta without inactivation and exposes the fetus. Increasing glucocorticoid dose to suppress follicular phase progesterone is desired, as progesterone accumulation is likely the greatest hormonal obstacle to fertility [17, 42]. For NCCAH, infertility is an indication for glucocorticoid therapy, and prednisone 4 – 5 mg/day in two divided doses can be given and increased to 7.5 mg/day if needed. In women with classic CAH, optimal glucocorticoid management should be attempted and may include dividing doses throughout the day or using a longer acting glucocorticoid (prednisone or prednisolone) for maximal reduction of androgen and progesterone. For both classic and NCCAH, if ovulation is not achieved by glucocorticoid treatment alone, it is reasonable to consider ovulation induction[43–49] with clomiphene citrate (only three case reports in the literature) [43, 50, 51], aromatase inhibitors, injectable gonadotropins, and adjuvant metformin [46, 47], however there is a paucity of data.

If refractory to these treatments, or in couples trying to avoid having a child with classic CAH, care with a REI specialist to utilize *in vitro* fertilization (IVF) can be offered, with or without preimplantation genetic testing for monogenic disorders (PGT-M). Since CAH is an autosomal recessive disorder, IVF and PGT-M is beneficial in cases where both parents are carriers of a classic *CYP21A2* mutation, one parent is a classic carrier and the other is affected, or a couple has a prior classic CAH-affected child. Some studies advocate that molecular testing for CAH should be routinely considered at fertility centers because of the high frequency, potential severity of disease of having an affected child with classic CAH, and the NCCAH association with reduced fertility [52]. Typically, embryos are created by combining oocytes (after controlled ovarian hyperstimulation and egg retrieval) with sperm and growing embryos to a day 5–6 blastocyst, at which point a biopsy of the trophectoderm (5–6 cells) is performed [53]. The trophectoderm is what becomes the placenta and this avoids disrupting the inner cell mass, which becomes the fetus. PGT-M can identify which embryos are affected vs. carriers for CAH (Fig. 2 and 3). In current practice, all embryos are typically frozen while PGT-M is performed via linkage analysis, single nucleotide polymorphism (SNP) arrays or next generation sequencing (NGS) to create a case specific probe. This often requires DNA samples from first degree relatives such as a grandparent or sibling or other embryos (including aneuploid ones) and can take approximately 6–8 weeks for the embryology lab to create a custom set-up for the couple [54, 55]. Then the chosen embryo is transferred in a subsequent cycle (Fig. 4).

If resistant to medical therapy, though controversial, an alternative treatment in select patients may include laparoscopic bilateral adrenalectomy. Though the Endocrine Society Clinical Practice Guidelines for CAH recommend against it [5], infertility may be a special indication for this surgery. In a meta-analysis for 48 cases in 32 studies of bilateral adrenalectomy, three of the 35 women underwent the surgery for primary infertility. All 3 women subsequently conceived, resulting in a total of 6 healthy live born babies [56–58]. Three additional patients underwent the surgery for obesity and virilization and all experienced spontaneous menses post-operatively [57]. However, bilateral adrenalectomy can increase the risk of future adrenal crises, which occurred in the three above mentioned patients as well as 8 of the 48 patients (17%) in another series [56, 57].

## Pregnancy in women with congenital adrenal hyperplasia

### Maternal risks and outcomes

Several studies have evaluated pregnancy outcomes in women with CAH [24, 59, 60]. A study in the United States using diagnosis codes in the Health Care Cost and Utilization Project-Nationwide Inpatient Sample database from 2004–2014, compared pregnancy outcomes in the general population and those with CAH. Unfortunately, this study was unable to distinguish between classic and non-classic CAH and medication data was not available. They found increased rates of chorioamnionitis, cesarean section, maternal infection, small for gestational age infants and congenital anomalies (which were not specified) but no increase in maternal hypertension or gestational diabetes [59]. In a series of pregnancy outcomes in patients with adrenal insufficiency, 32 women had classic CAH, and they were found to have a high rate of miscarriage (43.8%), cesarean section (64.3%) and preterm labor (32.3%). In this review, 2 of the 32 women had an adrenal crisis during pregnancy [60]. The most comprehensive data to date on pregnancy in women with CAH can be found in a Swedish population-based cohort of 272 women with CAH (69 gave birth to at least 1 child) [24]. Compared to controls matched by sex, age and place of birth, women with CAH had a higher rate of gestational diabetes (4.9% versus 1.4%) and cesarean section, (51.4% versus 12.3%). No differences were found in preeclampsia, infant Apgar score, and frequency of small-for-gestational age in the neonates. This cohort included 8 women with SW, 26 women with SV and 16 women with NCCAH. Outcomes between the different cohorts of CAH were equivalent except for the cesarean section rate which was 90.9%, 65.9% and 33.3% in these groups respectively, with the rate of 90% planned cesarean section for those with salt-wasting [24]. This higher rate of planned cesarean section in women with classic SW CAH is likely associated with the desire to avoid damage to the previous genital surgery in women born with a urogenital sinus [24].

### Options for prenatal diagnosis

As mentioned above preimplantation genetic testing can be performed during IVF. There are several options for prenatal diagnosis during pregnancy: non-invasive prenatal testing (maternal serum cell free-DNA) for sex of the baby (10 weeks—delivery), and chorionic villus sampling (CVS) at 10–13 weeks or amniocentesis (15–20 weeks) for diagnosis of CAH. Cell-free DNA is an analysis of circulating free fetal DNA collected from maternal blood; CVS is placental villi collected by a needle or special catheter transcervically or transabdominally under ultrasound guidance; amniocentesis is a collection of fetal cells obtained via a 22-gauge spinal needle, transabdominally from a pocket of amniotic fluid under ultrasound guidance [61].

In addition to identifying fetal chromosomal sex, one study accurately identified fetal CAH status using targeted massively parallel sequencing (MPS) of cell-free DNA in maternal plasma in 14 families as early as 5 weeks and 6 days gestation [62], however this is considered experimental. Cell-free DNA is currently offered and utilized as a screening test for chromosomes 13, 18, 21, X, and Y, where CVS and amniocentesis are considered diagnostic tests and are needed to definitively confirm most genetic disorders. When a fetus is identified as 46,XX based on cell-free DNA but male or atypical genitalia are identified on a second trimester anatomy ultrasound, amniocentesis for karyotype and *CYP21A2* mutation testing should be performed before assuming that the fetus has CAH, as a variety of conditions can explain these findings including testing errors [63]. It is also recommended that amniocentesis is performed for fetuses that were PGT-M tested embryos since the trophectoderm becomes the placenta, not the fetus, and CVS is a biopsy from the placenta, whereas amniocentesis analyzes fetal cells.

### Management during pregnancy

Based on the above limited information, we can conclude that women with CAH when they conceive have relatively successful pregnancies when compared to the general population. These pregnancies, however, are considered high risk given the complexity of medical management of CAH and comorbid conditions. It is thus important that care is coordinated between an obstetrician experienced in high-risk pregnancy and an endocrinologist experienced in the management of CAH [24]. Prenatal care should include routine monitoring for infections and preeclampsia. Although there are conflicting results regarding the risk of gestational diabetes, enhanced screening for gestational diabetes with glucose screen in the first or second trimester is prudent given that exposure to glucocorticoids may increase glucose intolerance.

Patients with classic CAH have been found to have a higher rate of chronic hypertension compared to the general population [64, 65], which along with chronic glucocorticoid use may increase the risk of intrauterine growth restriction (IUGR), which increases the risk for intrauterine fetal demise (IUFD). Often patients with CAH have comorbidities which include diabetes and obesity [65], which also might affect fetal growth [66]. Along with these theoretic risks, at least one case series of women with CAH reported increased risk of having a small for gestational age infant [59]. It is therefore reasonable to perform serial fetal growth ultrasounds starting in the third trimester. Although a higher incidence of fetal demise has not been found, studies to date comprise of small numbers, and are retrospective [24, 59, 60]. Thus, fetal wellbeing monitoring starting in the late third trimester should be performed given the high-risk nature of these pregnancies. If IUGR is detected, then standard antenatal fetal surveillance management is indicated, and timing of delivery is determined by umbilical artery doppler findings [67, 68].

Finally, women with CAH may be at increased risk of infection. Labor and delivery are associated with high rates of infection under normal circumstances, and increased risk of infection was found in pregnant women with CAH in a U.S. retrospective population-based study [59]. Thus, a high index of suspicion for chorioamnionitis should be maintained, and women with CAH should be treated with antibiotics if there is prolonged rupture of membranes, maternal temperature reaches 100.4 during labor, and prophylactically prior to incision when a cesarean section is undertaken [69, 70].

## Glucocorticoid and mineralocorticoid therapy

### Preconception

Treatment of classic CAH includes the administration of lifetime glucocorticoid and usually mineralocorticoid therapy. As stated previously, hydrocortisone and prednisolone/prednisone are preferred as they are both inactivated by 11β-hydroxysteroid dehydrogenase type 2 in the placenta and thus prevent fetal exposure. Dexamethasone, however, does cross the placenta and should be avoided during pregnancy unless the purpose is to expose the fetus (such as in the case of administration for fetal lung maturation when pre-term delivery is expected). Efficient placental aromatization of maternal androgens prevents the female fetus from virilization, thus maternal CAH is not likely to lead to in-utero fetal virilization of an unaffected female [24, 71]. If glucocorticoid therapy is initiated to treat subfertility in a woman with NCCAH, it is typically continued throughout pregnancy, but data is limited. In this cohort, the miscarriage rate can be reduced to normal in women treated with glucocorticoid prior to and during pregnancy [21].

### During pregnancy

Steroid management during pregnancy requires careful consideration. During normal pregnancy, there is a progressive increase in circulating corticotropin-releasing hormone (CRH) and adrenocorticotropic hormone (ACTH), and the estrogen-stimulated increase in sex hormone–binding globulin and corticosteroid-binding globulin cause androgen and cortisol levels to naturally increase. In addition, free cortisol increases after the second trimester [72]. It is therefore reasonable to expect that women with CAH will require an increase of their treatment dose as the pregnancy progresses. Our practice is to increase glucocorticoid dose by approximately 20% in the second or third trimester. This is in agreement with the Endocrine Society Clinical Guidelines’ suggestion that women should be maintained on their pre-pregnancy doses of glucocorticoid and mineralocorticoid, and doses of glucocorticoid should be increased by 20% to 40% from the 24th week [5, 73]. However, there are no evidence-based recommendations for steroid management during pregnancy.

Typical biomarkers such as 17-hydroxyprogesterone, androstenedione and plasma renin activity change physiologically with pregnancy. Normal ranges during pregnancy are not available and therefore these biomarkers cannot be used in the management of the pregnant woman with CAH. Free testosterone levels can be measured to target levels in the high normal range for pregnancy [74].

Care should be given to avoid cushingoid side effects from too high a dose of glucocorticoid.

Conversely, cases of adrenal crisis in pregnancy have been reported, but symptoms of adrenal insufficiency such as postural hypotension and fatigue are common during normal pregnancy [60]. It is, therefore, important to educate the patient regarding stress dosing for intercurrent illnesses, and maintain a high index of suspicion for possible adrenal insufficiency (see below). Patients should be provided an emergency hydrocortisone injection set and receive training for self-injection.

Doses of fludrocortisone may need to be increased during pregnancy as progesterone and 17-hydroxyprogesterone levels rise during pregnancy and compete with mineralocorticoid receptors. In general, hydrocortisone will exert some mineralocorticoid activity which may compensate for the need for higher fludrocortisone [49]. Plasma renin activity physiologically increases during pregnancy, [75] but treatment can be monitored through blood pressure and serum sodium and potassium [73], However, it should be noted that normative ranges may be altered during pregnancy [75]. For example, serum sodium reference ranges for pregnant women are 2–5 nmol/L lower than nonpregnant women probably due to increased glomerular filtration rate (GFR)[76]. Clinical monitoring for general wellbeing, blood pressure, and weight gain during pregnancy is essential. In principle, management of glucocorticoid and mineralocorticoid replacement is based on clinical grounds as no reliable laboratory-based assessment has been established.

### Prenatal treatment

Importantly, maternal dexamethasone started in the first trimester prior to testing for the chromosomal sex of the baby and administered in order to prevent virilization of a female fetus, is no longer recommended [5, 77]. Giving dexamethasone before week 9 of gestation can suppress fetal adrenal androgen production and prevent virilization of the external female genitalia during the period of urogenital organogenesis. This approach has been shown to be successful in ameliorating virilization of the external genitalia in studies in the U.S. and Europe. [62, 78–80] However, routine administration will more often expose unaffected fetuses (seven out of eight) including unaffected and heterozygous females as well as males, who are not at risk for atypical genitalia. Given exposure of unaffected fetuses, the concern of glucocorticoid effects on fetal brain development and postnatal growth [48], the unknown long term side-effects in children, and ethical concerns, Endocrine Societies recommend that prenatal treatment is only performed as part of Institutional Review Board approved studies [5, 77]. There are no studies in the U.S. currently evaluating this.

### Adrenal crisis

It is unknown whether pregnancy is associated with an increased risk of adrenal crisis in women with CAH, but cases have been reported. It is important to review with the patient and family the requirements for higher doses of glucocorticoid during illnesses, similar to “sick day rules” in the nonpregnant state, [81] but which may include episodes of hyperemesis [49]. If a pregnant woman experiences an adrenal crisis, she requires urgent attention with administration of stress dose steroids and intravenous fluids in a manner similar to the nonpregnant state [82]. If the pregnancy has entered the period of fetal viability (after 24 weeks gestational age) when an adrenal crisis is encountered, it is important that fetal monitoring for wellbeing and contractions be performed during maternal treatment. In such cases the patient should be co-managed by an endocrinologist experienced in CAH care as well as a high-risk obstetrician [67, 68].

### During labor and delivery

Labor is a “stress” event equivalent to major surgery and thus requires the administration of stress dose steroids. Hydrocortisone should be initiated with the onset of active labor with an intravenous bolus injection of 100 mg hydrocortisone followed by 50 mg of hydrocortisone every 6 h or a continuous infusion of 200 mg hydrocortisone/24 h throughout labor and delivery (Cesarean section or vaginal delivery). Intravenous fluids are also given, and close cardiovascular monitoring is indicated.

## Post-partum care and lactation

Following delivery, stress dose steroids are given and quickly weaned over the next few days. In general, the predelivery glucocorticoid dose should be doubled for 24–48 h and then tapered quickly to the pre-pregnancy dose. It is important for the patient’s endocrinology team to be consulted early in the post-partum period to evaluate the patient as doses are weaned to pre-pregnancy levels [49]. Evaluation of adrenal function in the newborn is not necessary. However, it is important that all newborns be screened for CAH according to the neonatal screening program in their country of birth. As women with CAH receive chronic glucocorticoid treatment, they may be at risk for poor wound healing and infections. Thus, surgical sites and tears should be monitored closely to assure proper healing. Overall, proposed management of CAH during before, during, and after pregnancy is summarized in Table 2.

**Table 2:** Proposed Management of Congenital Adrenal Hyperplasia (CAH) During Pregnancy

**Trimester**	**Classic CAH**	**Nonclassic CAH**
Preconception	Initiate prenatal vitamins with folic acid for neural tube protection Optimize GC treatment to achieve ovulation Offer partner carrier testing if not done Referral to REI if partner is a carrier	Initiate prenatal vitamins with folic acid for neural tube protection Initiate GC for infertility or history of miscarriage: prednisone 4—5 mg/day in two divided doses. Increase to 7.5 mg/day as needed for ovulation Offer partner carrier testing if not done Referral to REI if difficulty conceiving
First Trimester (Up to 12 weeks from LMP)	Routine prenatal laboratory testing Early glucose screening for gestational diabetes Continue prenatal GC therapy Counsel about stress dosing Monitor for adrenal crisis (especially during episodes of hyper-emesis) May be offered: Non-invasive prenatal testing (cell-free DNA) ----for fetal sex chromosomes and comparison to external genitalia on ultrasound Chorionic villus sampling (10–13 weeks)	Routine prenatal laboratory testing Early glucose screening for gestational diabetes Continue GC therapy if initiated prenatally Offer partner carrier testing May be offered: Non-invasive prenatal testing (cell-free DNA) ----for fetal sex chromosomes and comparison to external genitalia on ultrasound Chorionic villus sampling (10–13 weeks)
Second Trimester (12–24 weeks from LMP)	Routine second trimester testing Glucose screening if not done in the first trimester May be offered: Amniocentesis (15–20 weeks) Monitor for adrenal crisis	Routine second trimester testing Continue GC treatment Glucose screening if not done in the first trimester May be offered: Amniocentesis (15–20 weeks) Monitor for adrenal crisis if receiving GC therapy
Third Trimester (24–41 weeks from LMP)	GC dose should be increased by 20–40% from the 24th week Routine prenatal laboratory testing including repeat glucose screening if previous one was normal Monthly fetal growth ultrasound Begin weekly fetal monitoring at 36 weeks (earlier if adrenal crisis or fetal growth restriction)	Routine prenatal laboratory testing including repeat glucose screening if previous one was normal Continue GC treatment Fetal growth scans and fetal monitoring based on usual indications (such as maternal diabetes)
Delivery	Planned cesarean section if history of total urogenital mobilization surgery Stress dose steroids Monitor for infection	Stress dose steroids if receiving glucocorticoid therapy during pregnancy
Postpartum	Wean GC to pre-pregnancy dose Monitor for infection and wound healing Discuss family planning options and desires	Wean GC Monitor for infection and wound healing Discuss family planning options and desires

*CAH* congenital adrenal hyperplasia, *GC* glucocorticoid, *LMP* last menstrual period

Corticosteroids are closely bound to proteins and both prednisolone and prednisone have been shown to be excreted in low levels in breast milk [83–85] with infant doses relative to the mother’s of 0.35% to 0.53% and 0.09% to 0.18%, for prednisone and prednisolone respectively. Infants are naturally exposed to maternal glucocorticoids during breastfeeding and thus it is not clear whether infant exposure to the exogenous maternal glucocorticoids in CAH is indeed undesired [86]. Thus given low rates of exposure and natural exposure to maternal glucocorticoids, as well as the known benefits of breast milk for infants, women with CAH can be encouraged to breastfeed [87]. Useful resources for the clinician prescribing medications during lactation include the websites Infant Risk Center at Texas Tech University Health Sciences Center [88] and Drugs and Lactation Database (LactMed) [89]. It is unknown whether women with CAH have altered lactation or difficulty with breastfeeding. Lastly, post-partum women with CAH can be offered contraception, but it should be noted that combined hormonal contraceptives are not recommended in the first 3 weeks post-partum and in breastfeeding women [16].

## Summary

This review highlights reproductive health in women with CAH due to 21-hydroxylaze deficiency including genetic transmission, contraception options, and fertility treatments, as well as glucocorticoid management during pregnancy, delivery, and postpartum. In our opinion, with genetic testing becoming more affordable and easily accessible, all patients with clinically diagnosed CAH should be offered genetic testing to determine their specific variant. If fertility is desired, then partners should have carrier testing and referral to a genetics counselor to assess the risk of having an affected child and discuss options such as IVF with PGT-M to select unaffected embryos if indicated and desired. During pregnancy, women with classic CAH should be followed closely by high-risk obstetricians in collaboration with an endocrinologist.

There are many unanswered questions around reproductive health for CAH due to 21-hydroxylase deficiency, a rare disease, though common in its mild (nonclassic) form. Further research is needed regarding contraception preferences, contraceptive side effects and effectiveness, fertility outcomes including rates of pregnancy on glucocorticoid alone and with ovulation induction agents, and IVF fetal and maternal outcomes. Prospective studies are needed to better evaluate treatments and outcomes as many women with CAH desire fertility and pregnancy.

## Abbreviations

GC: Glucocorticoid
IVF: *In vitro* fertilization
PGT-M: Preimplantation genetic testing monogenic disorders
